# Drought–salinity interaction suppresses seed germination and seedling growth in alfalfa by altering water status, membrane stability, and antioxidant responses

**DOI:** 10.3389/fpls.2026.1887737

**Published:** 2026-07-28

**Authors:** Yuanzhen Zhou, Tianpeng Li, Xinyue Zhu, Binbin Wang, Zhenggang Guo, Minguo Liu, Jing Zhang

**Affiliations:** 1 College of Grassland Science, Gansu Agricultural University, Lanzhou, China; 2 State Key Laboratory of Herbage Improvement and Grassland Agro-ecosystems, Engineering Research Center of Grassland Industry, Ministry of Education, College of Pastoral Agriculture Science and Technology, Lanzhou University, Lanzhou, China; 3 College of Grassland Agriculture, Northwest A&F University, Yangling, Shaanxi, China

**Keywords:** alfalfa, antioxidant enzymes, drought-salinity, membrane stability, seed germination, seedling growth, water status

## Abstract

Drought and salinity stress are the primary limiting factors for the successful establishment of alfalfa (*Medicago sativa*) pastures. However, the integrated responses of alfalfa during early establishment to combined drought and salinity stress, particularly the physiological links among water status, membrane stability, antioxidant defense, seed germination, and seedling growth, remain insufficiently understood. This study employed a two-factor design consisting of PEG-6000-induced drought stress at three levels (5%, 15%, and 25%) and NaCl-induced salinity stress at three concentrations (50, 100, and 150 mmol·L^-1^). The experiment included nine drought–salinity stress combinations plus a non-stress control. The interaction between drought and salinity stress significantly affected alfalfa seed germination and seedling physiological traits. Increasing PEG and NaCl concentrations generally reduced seed germination indices, including germination percentage, germination potential, germination index, and vigor index, and inhibited seedling growth, as reflected by decreases in plant height, leaf number, fresh weight, and aboveground dry weight. Combined drought–salinity stress also disrupted water status and membrane stability, increased relative electrical conductivity, and induced stress-specific changes in antioxidant enzyme activities. Severe combined stress caused the strongest inhibition, whereas low-intensity combined stress treatments, especially 5% PEG combined with 50 or 100 mmol·L^-1^ NaCl, showed relatively better overall performance among the imposed stress treatments under controlled conditions. These findings provide a physiological basis for evaluating early-stage alfalfa responses to combined drought and salinity stress and may support future screening of stress-tolerant germplasm.

## 1 Introduction

Alfalfa (Medicago sativa L.), commonly known as the “king of forages”, is one of the most important perennial leguminous forage crops. Owing to its high protein content, good palatability, and strong biological nitrogen-fixing capacity, alfalfa plays an irreplaceable role in animal husbandry and sustainable forage production (Zhang and Wang, 2025; Desta, 2026b). In addition, its well-developed root system can effectively improve soil structure, enhance soil fertility through biological nitrogen fixation, and reduce soil erosion, indicating considerable potential for ecological restoration and soil sustainability (Desta, 2026a, b). In recent years, with the rapid increase in demand for high-quality forage in China, the planting area of alfalfa has continued to expand. However, constrained by the national policy of maintaining 1.8 billion mu of arable land, alfalfa production has gradually shifted toward marginal lands in Northwest and North China. These regions are frequently characterized by limited water availability and soil salinization, which jointly restrict alfalfa establishment, early growth, and subsequent productivity (Wan et al., 2023). Therefore, improving alfalfa establishment under drought–salinity stress has become a critical issue for sustainable forage production in marginal environments. Because alfalfa production is increasingly expanding into arid and saline marginal areas, understanding how early establishment responds to drought and salinity stress is essential for improving pasture formation under adverse environments. Seed germination and early seedling growth are the most vulnerable stages of alfalfa establishment. Together, these two stages determine seedling emergence, stand formation, and the initial development of productive pasture swards. Successful germination requires rapid water uptake, activation of respiration, reserve mobilization, enzyme activity, and radicle protrusion (Carrera-Castaño et al., 2020; Upretee et al., 2024). After germination, early seedling growth, including leaf expansion, stem elongation, root development, and biomass accumulation, further determines whether newly emerged seedlings can survive and establish under adverse environments. At these early stages, drought and salinity stress directly interfere with water uptake, metabolic activation, and early tissue formation, thereby reducing germination performance and seedling vigor (Chaves et al., 2009; Arif et al., 2020; Liu et al., 2022). Salinity-induced inhibition of seed germination is generally associated with reduced imbibition, osmotic stress, Na^+^/Cl^-^ toxicity, oxidative damage, membrane injury, and altered hormonal balance between abscisic acid and gibberellins (Acosta-Motos et al., 2017; Manono, 2026). Because alfalfa seeds are small and early seedlings have limited stress-buffering capacity, even moderate drought or salinity may cause disproportionate reductions in seedling emergence, early seedling growth, and stand establishment. Thus, identifying the response patterns of germination and early seedling traits under drought and salinity stress is essential for improving alfalfa establishment in stress-prone areas.

A substantial body of research has examined the individual effects of drought or salinity on alfalfa germination, seedling growth, and physiological metabolism. Drought stress mainly reduces external water potential, restricts water uptake, and decreases cellular turgor, whereas salinity stress imposes both osmotic stress and ion toxicity through excessive Na^+^ and Cl^-^ accumulation. Although these two stresses differ in their primary causes, both can impair cell division and elongation, disrupt membrane integrity, increase electrolyte leakage, and induce oxidative damage when antioxidant defense is insufficient (Chaves et al., 2009; Angon et al., 2022; Chandran et al., 2024). These studies have greatly improved our understanding of single-stress responses in alfalfa. However, field environments rarely impose drought or salinity in isolation, particularly in arid and saline–alkali regions, where drought stress and salt accumulation often occur simultaneously. Combined drought and salinity stress may generate more complex plant responses than either stress alone (Sahoo et al., 2025). Under combined stress, plants simultaneously experience restricted water uptake, osmotic imbalance, ion toxicity, membrane injury, and oxidative stress, which may interact additively, synergistically, or antagonistically (Zhou Z. et al., 2024; Angon et al., 2022; Cao et al., 2023). Recent studies and reviews have suggested that drought–salinity interactions can strongly alter water status, membrane stability, photosynthetic function, ion regulation, and antioxidant defense. Nevertheless, the integrated responses of alfalfa during the early establishment stage remain insufficiently understood. Specifically, it remains unclear how combined drought and salinity stress, rather than either stress alone, coordinately regulates seed germination, seedling growth, water status, membrane stability, and antioxidant enzyme activity during early alfalfa establishment. In particular, most previous studies have focused on either adult plants or single-stress treatments, whereas fewer studies have systematically evaluated how combined drought and salinity regulate germination performance, seedling growth, leaf water status, membrane stability, and antioxidant enzyme activities during the transition from seed germination to early seedling growth.

In this study, we investigated the combined effects of drought and salinity stress on seed germination, seedling growth, and physiological responses of alfalfa through laboratory Petri dish assays and pot experiments. The main objectives were: (1) to analyze the responses of alfalfa seed germination characteristics and seedling growth traits to different drought and salinity stress combinations; (2) to clarify the changes in leaf water status, membrane stability, and antioxidant enzyme activities under combined drought and salinity stress; and (3) to identify the key indicators and suitable stress thresholds associated with alfalfa tolerance or injury during early establishment. We hypothesized that combined drought and salinity stress would suppress seed germination and seedling growth by disrupting water status, membrane stability, and antioxidant defense, and that high-intensity drought–salinity combinations would cause stronger injury than low-intensity combinations. The findings of this study are expected to provide a physiological basis for evaluating alfalfa tolerance to combined drought and salinity stress and to support the establishment and cultivation of alfalfa in arid and saline–alkali regions.

## 2 Materials and methods

### 2.1 Experimental materials

The plant material used in this study was *Medicago sativa* L. ‘Gannong No. 3’, a widely cultivated alfalfa cultivar in northwestern China with strong adaptability, good regrowth capacity, and relatively high biomass productivity. The seeds were provided by the College of Grassland Science, Gansu Agricultural University.

### 2.2 Experimental design

The experiment was conducted from June to August 2024 in a controlled growth chamber at the College of Grassland Science, Gansu Agricultural University. The growth chamber was maintained at 25 ± 1 °C, with a relative humidity of 60%–70%, a 16 h/8 h light/dark photoperiod, and a photosynthetic photon flux density of approximately 180 μmol·m^-^²·s^-1^.

A two-factor experiment was arranged in a completely randomized design. The first factor was drought stress, simulated using PEG-6000 solutions at three concentrations: 5%, 15%, and 25% (w/v). The second factor was salinity stress, imposed using NaCl solutions at three concentrations: 50, 100, and 150 mmol·L^-1^. The combination of the two factors generated nine drought–salinity stress treatments. In addition, a non-stress control treatment was established using distilled water without PEG-6000 or NaCl. Thus, the experiment included ten treatments in total, and each treatment was replicated five times.

Because the osmotic potentials of the treatment solutions were not directly measured, they were estimated to better characterize the physiological intensity of the imposed drought–salinity treatments. The osmotic potential of PEG-6000 solutions was estimated using the empirical equation of Michel and Kaufmann (1973), and the osmotic contribution of NaCl was estimated using the van’t Hoff equation. For PEG-6000, concentrations of 5%, 15%, and 25% (w/v) were approximately converted to 50, 150, and 250 g·kg^-1^ water, respectively. For NaCl, ideal dissociation was assumed, with a van’t Hoff factor of 2. The combined osmotic potential of each PEG–NaCl mixture was approximated as the sum of the PEG-induced osmotic potential and the NaCl-induced osmotic potential. These values were used only as estimated reference values to distinguish generalized osmotic stress from the additional ionic component imposed by NaCl. The estimated osmotic potentials of all treatment solutions are shown in **Supplementary Table 1**.

For the seed germination experiment, healthy and uniform alfalfa seeds were selected and surface-sterilized before treatment. Fifty seeds were placed in each 9-cm Petri dish lined with two layers of filter paper. An equal volume of the corresponding PEG–NaCl mixed solution was added to each dish according to the treatment design, while distilled water was added to the control dishes. The dishes were randomly placed in the growth chamber, and their positions were changed regularly to minimize possible positional effects. Evaporative water loss was replenished daily to maintain a constant solution volume. Filter papers were replaced every 72 h, and mold-contaminated seeds were removed. The germination experiment was terminated on day 10.

For the seedling growth experiment, seeds were sown in pots filled with a mixed substrate of vermiculite and perlite at a ratio of 1:1 (v/v). Each pot initially contained 20 seeds and was supplied with 100 mL Hoagland nutrient solution every 48 h before stress treatment. When the seedlings reached the trifoliate stage, they were thinned to six uniform plants per pot. The drought–salinity treatments were then applied according to the same PEG–NaCl treatment design described above, with distilled water used for the control treatment. During the 20-day stress period, the corresponding PEG–NaCl treatment solutions were applied directly to the pot substrate as irrigation solutions at the same volume and interval for all treatments. Solution volume was adjusted every 48 h using the corresponding treatment solution to maintain comparable water supply among treatments and to reduce dilution effects. Pot positions were changed regularly to minimize positional effects in the growth chamber. Substrate electrical conductivity and PEG concentration in the pot medium were not continuously monitored during the stress period; therefore, the nominal PEG and NaCl concentrations were used to represent the imposed treatment levels.

### 2.3 Measurement and calculation

#### 2.3.1 Determination of seed germination parameters

During the 10-day germination period, the number of germinated seeds was recorded daily. Germination was defined as visible radicle protrusion through the seed coat. Germination parameters were calculated according to the ISTA rules and previously described methods with minor modifications (ISTA, 2018; Upretee et al., 2024). Germination percentage and germination potential were calculated using Equations 1 and 2, respectively. Germination potential was calculated on day 3, and germination percentage was calculated on day 10, using the following equations:

(1) $$\begin{array}{c} \mathrm{Germination percentage}\;(\%)=\frac{n_{10}}{N}\times 100 \end{array}$$

(2) $$\begin{array}{c} \mathrm{Germination potential}\;(\%)=\frac{n_{3}}{N}\times 100 \end{array}$$

where n_10_ is the number of germinated seeds on day 10, n_3_ is the number of germinated seeds on day 3, N is the total number of tested seeds.

The germination index and vigor index were calculated using Equations 3 and 4, respectively. The germination index (*GI*) and vigor index (*VI*) were calculated as follows:

(3) $$\begin{array}{c} GI=\sum \frac{G_{t}}{D_{t}} \end{array}$$

(4) $$\begin{array}{c} VI=GI\times S \end{array}$$

where *Gt* denotes the number of seeds germinated on day t, *Dt* denotes the corresponding germination day, and *S* represents the mean radicle length.

#### 2.3.2 Determination of seedling growth parameters

After 20 days of stress treatment, alfalfa seedlings were randomly selected from each treatment for the determination of growth parameters. For each biological replicate, seedlings from the same pot were used for growth and biomass measurements. Plant height (cm) was measured from the stem base to the highest point of the plant using a ruler. The number of leaves was recorded using seedlings with uniform growth, counting from the first true leaf at the stem base. Fresh weight (g) was determined by cutting the aboveground parts of the seedlings evenly at the surface of the potting substrate and immediately weighing them. For aboveground dry weight (g), the harvested aboveground tissues were rinsed thoroughly, heated at 105 °C for 30 min to deactivate enzymatic activity, and then oven-dried at 80 °C to constant weight before being weighed using an analytical balance.

#### 2.3.3 Determination of seedling physiological parameters

After growth measurements, fresh seedling samples were collected for physiological analyses and stored at 4 °C before measurement. Fresh samples for water content, relative electrical conductivity, and antioxidant enzyme assays were collected from the corresponding biological replicate after growth measurements. When the sample amount was insufficient for all physiological determinations, additional parallel seedlings from the same pot were used. Tissue water content was determined using the oven-drying method, following the method described by Ma et al. (2017). Relative electrical conductivity was measured using a conductivity meter. Briefly, plant samples were immersed in deionized water, and the initial electrical conductivity (*C_1_*) was recorded. The samples were then subjected to a boiling-water bath, after which the final electrical conductivity (*C_2_*) was measured. Relative electrical conductivity was calculated according to Equation 5.

(5) $$\begin{array}{c} \mathrm{Relative electrical conductivity}\;(\%)=\;\frac{C1}{C2}\;\times \;100 \end{array}$$

The activities of superoxide dismutase (SOD), peroxidase (POD), and catalase (CAT) were determined using commercial assay kits from Beijing Solarbio Science & Technology Co., Ltd. (BC0170, BC0090, and BC0200, respectively). SOD and POD activities were measured by visible spectrophotometry at 550 nm and 470 nm, respectively, whereas CAT activity was determined by ultraviolet spectrophotometry at 240 nm. All procedures were performed strictly according to the manufacturer’s standardized protocols.

### 2.4 Statistical analyses

Prior to statistical analysis, the normality and homogeneity of variance of each variable were tested using the Shapiro–Wilk test and Levene’s test, respectively. When necessary, data were transformed to meet the assumptions of analysis of variance. For the nine drought–salinity stress treatments, two-way analysis of variance (ANOVA) was performed to examine the main effects of drought stress (D), salinity stress (S), and their interaction (D × S) on seed germination, seedling growth, and physiological traits. In addition, to compare all treatments including the non-stress control, one-way ANOVA followed by Tukey’s honestly significant difference (HSD) test was conducted. Differences were considered significant at *P* < 0.05 and highly significant at *P* < 0.001.

Pearson correlation analysis was performed to evaluate the relationships among seed germination traits, seedling growth traits, water-related traits, membrane stability, and antioxidant enzyme activities. A correlation heatmap was generated to visualize the direction and strength of trait associations. Before principal component analysis (PCA), all measured variables were standardized using Z-score transformation to eliminate the influence of different units and scales. PCA was then conducted to identify the major dimensions of variation among treatments and to determine the traits contributing most strongly to treatment separation.

The membership function method was used to comprehensively evaluate the overall performance of alfalfa under different drought–salinity stress combinations. Germination percentage, germination potential, germination index, vigor index, plant height, leaf number, fresh weight, aboveground dry weight, and leaf water content were treated as positive performance indicators, whereas relative electrical conductivity was treated as a negative injury indicator. Antioxidant enzyme activities, including SOD, CAT, and POD, were considered stress-response indicators rather than uniformly positive performance traits, because elevated enzyme activity may reflect either enhanced antioxidant defense or greater oxidative stress. Therefore, a sensitivity analysis was further conducted by excluding SOD, CAT, and POD from the membership function evaluation to test whether antioxidant enzyme scoring affected the treatment ranking. Positive and negative membership functions were calculated using Equations 6 and 7, respectively. Positive indicators, including germination percentage, germination potential, germination index, vigor index, plant height, leaf number, fresh weight, aboveground dry weight, and leaf water content, were standardized using the positive membership function:

(6) $$\begin{array}{c} U(X_{ij})=\;\frac{\left(X_{ij}-Xmin\right)}{Xmax-Xmin} \end{array}$$

Negative indicators, such as relative electrical conductivity, were standardized using the negative membership function:

(7) $$\begin{array}{c} U(X_{ij})=\frac{\left(Xmax-\;X_{ij}\right)}{\left(Xmax-Xmin\right)} \end{array}$$

where X_ij_ represents the value of the j-th trait under the i-th treatment, and Xmax and Xmin represent the maximum and minimum values of the corresponding trait, respectively. The average membership value of all evaluated traits was calculated for each treatment and used to rank the comprehensive performance of alfalfa under combined drought and salinity stress.

Structural equation modeling (SEM) was conducted as an exploratory analysis to examine potential associations among stress factors, intermediate trait groups, seed germination, and aboveground dry weight. The SEM was based on biological replicate-level data, n = 50. Drought stress and salinity stress were treated as exogenous variables. Growth traits, water-related traits, and antioxidant enzyme traits were included as intermediate trait groups, while seed germination and aboveground dry weight were used as final response variables. Seed germination was represented by the vigor index, which was selected based on the coefficient of variation among germination-related traits. All observed variables were standardized before model construction to reduce the influence of different units and scales. Standardized path coefficients were used to describe the strength and direction of associations among variables. Model fit was assessed using the chi-square test, the ratio of chi-square to degrees of freedom (χ²/df), comparative fit index (CFI), Tucker–Lewis index (TLI), root mean square error of approximation (RMSEA), and standardized root mean square residual (SRMR). Because the experiment was conducted under controlled conditions and the SEM was exploratory, the model was interpreted as evidence of potential statistical associations rather than definitive causal pathways.

ANOVA and multiple comparisons were performed using SPSS 26.0 (SPSS Inc., Chicago, IL, USA). Correlation analysis, PCA, and membership function ranking plots were generated using Origin 2024 (OriginLab Inc., Northampton, MA, USA). Structural equation modeling was performed using the lavaan package in R.

## 3 Results

### 3.1 Combined drought and salinity stress suppresses alfalfa seed germination

**Figure 1:**
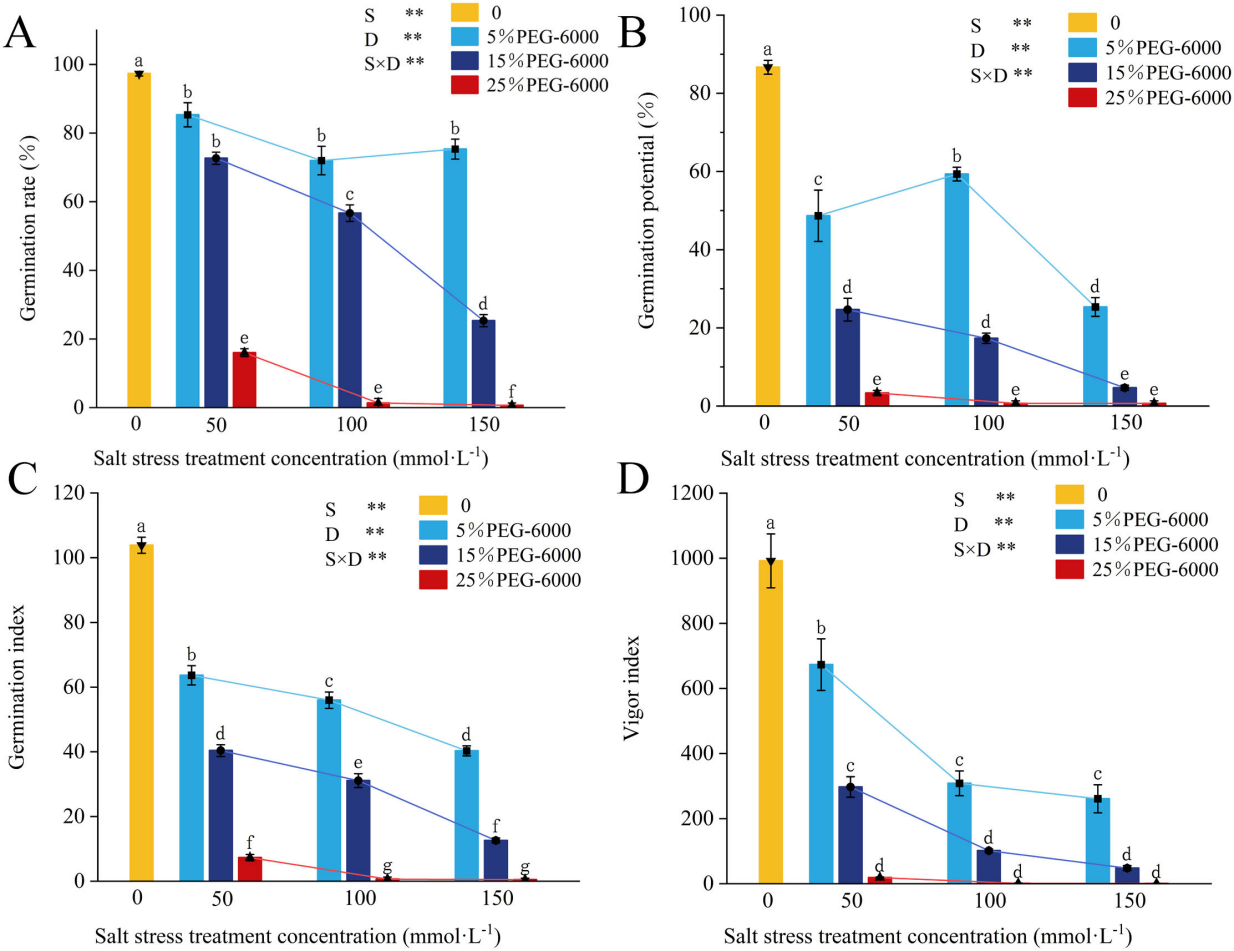
Effects of combined drought and salinity stress on the germination characteristics of alfalfa seeds. **(A)** Germination rate, **(B)** Germination potential, **(C)** Germination index, **(D)** Vigor index. D, drought stress; S, salinity stress; D × S, interaction between drought stress and salinity stress. Different lowercase letters above bars indicate significant differences among treatments at *P* < 0.05. *, **, and *** indicate significance at *P* < 0.05, *P* < 0.01, and *P* < 0.001, respectively; ns indicates no significant difference.

Drought stress, salinity stress, and their interaction all had highly significant effects on the germination percentage, germination potential, germination index, and vigor index of alfalfa seeds (*P* < 0.001; **Figure 1**). Under the same salinity level, the germination percentage, germination potential, germination index, and vigor index of alfalfa seeds consistently decreased as drought stress intensified. Under the same drought level, the germination index and vigor index also decreased continuously with increasing salinity intensity. For germination percentage, under mild drought stress, it first decreased and then increased as salinity stress intensified, whereas under moderate and severe drought stress, it decreased continuously with increasing salinity intensity. For germination potential, under mild drought stress, it first increased and then decreased with increasing salinity intensity, whereas under moderate and severe drought stress, it continuously decreased as salinity stress intensified.

### 3.2 Seedling growth is constrained by increasing drought–salinity intensity

#### 3.2.1 Plant height and leaf number

**Figure 2:**
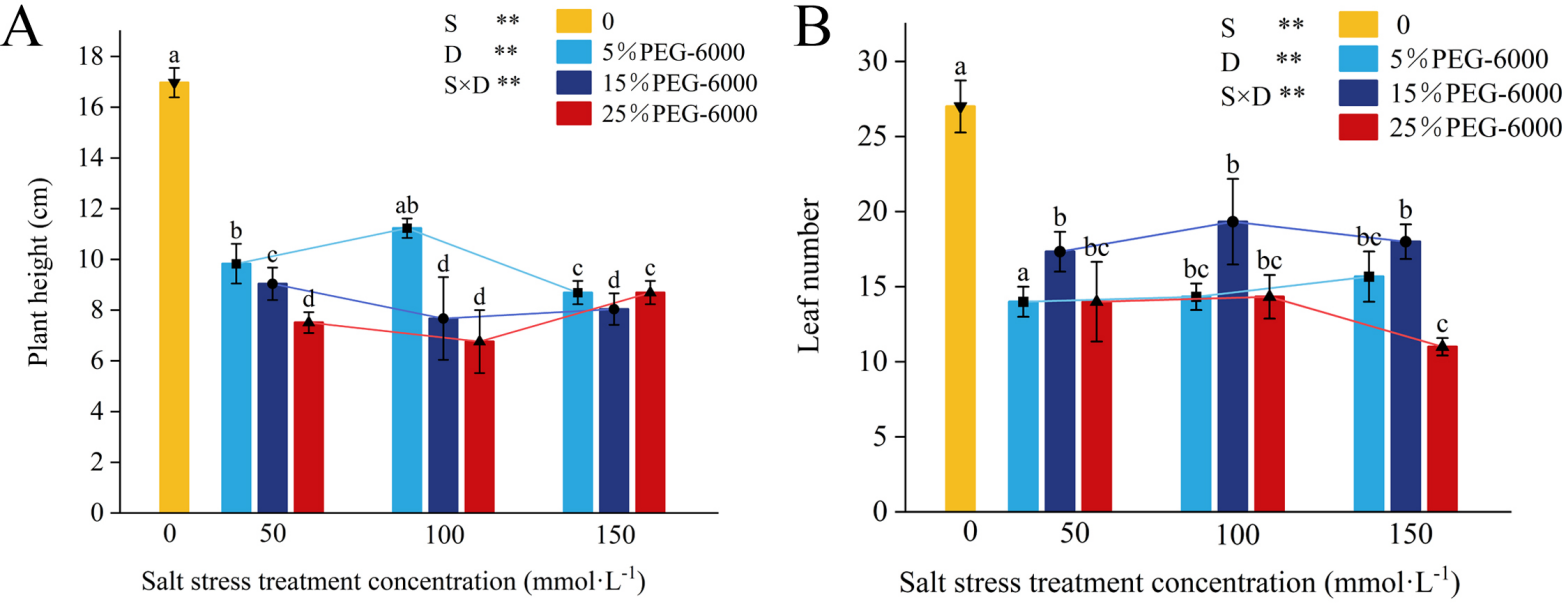
Effects of combined drought and salinity stress on the number of leaves and plant height of alfalfa seedlings. **(A)** Plant height, **(B)** Leaf number. D, drought stress; S, salinity stress; D × S, interaction between drought stress and salinity stress. Different lowercase letters above bars indicate significant differences among treatments at *P* < 0.05. *, **, and *** indicate significance at *P* < 0.05, *P* < 0.01, and *P* < 0.001, respectively; ns indicates no significant difference.

Drought stress, salinity stress, and their interaction had highly significant effects on plant height and leaf number in alfalfa seedlings (*P* < 0.001; **Figure 2**). Under the same NaCl concentration gradient, the number of leaves in alfalfa seedlings first increased and then decreased as the level of drought stress increased. Under moderate and severe drought-stress conditions, leaf number also first increased and then decreased with increasing salinity intensity and reached the lowest value of 12 leaves under the 25% PEG + 150 mmol·L^-1^ NaCl treatment. Under mild drought-stress conditions, however, leaf number showed a gradual increase with increasing salinity intensity. Under mild and moderate salinity-stress conditions, plant height in alfalfa seedlings decreased as drought stress intensified. Under severe salinity stress, however, plant height first decreased and then increased with increasing drought-stress intensity. Under moderate and severe drought-stress conditions, plant height first decreased and then increased slightly as the NaCl concentration gradient increased. In contrast, under mild drought-stress conditions, plant height first increased to a peak value of 11.23 cm and then decreased with increasing salinity intensity.

#### 3.2.2 Fresh weight and aboveground dry weight

**Figure 3:**
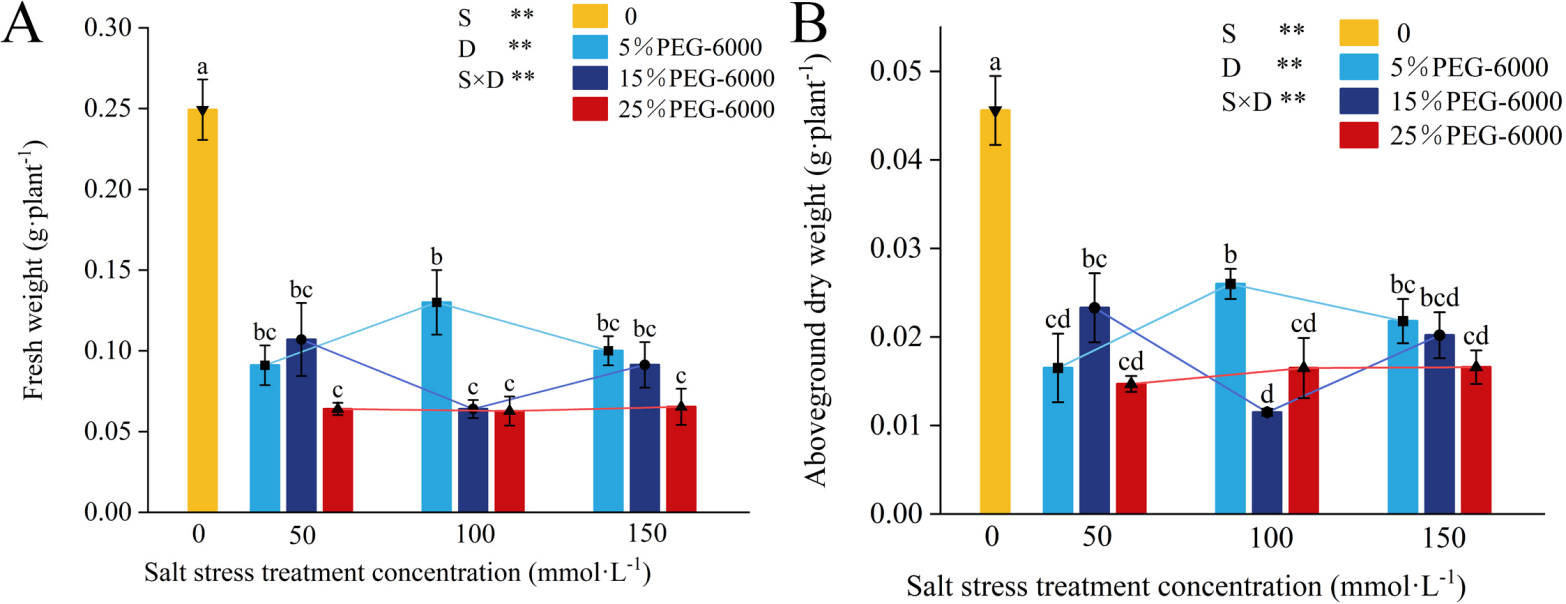
Effects of combined drought and salinity stress on the aboveground dry weight and fresh weight of alfalfa seedlings. **(A)** Fresh weight, **(B)** Aboveground dry weight. D, drought stress; S, salinity stress; D × S, interaction between drought stress and salinity stress. Different lowercase letters above bars indicate significant differences among treatments at *P* < 0.05. *, **, and *** indicate significance at *P* < 0.05, *P* < 0.01, and *P* < 0.001, respectively; ns indicates no significant difference.

Drought stress, salinity stress, and their interaction all had highly significant effects on the fresh weight and aboveground dry weight of alfalfa seedlings (*P* < 0.001; **Figure 3**). Under moderate and severe salinity stress, fresh weight gradually decreased with increasing drought intensity. By contrast, under mild salinity stress, fresh weight initially increased and then declined as drought intensity increased. Under moderate and severe drought stress, fresh weight first decreased and then increased with increasing NaCl concentration. Under mild drought stress, however, fresh weight initially increased to a maximum value of 0.13 g·plant^-1^ and subsequently decreased as NaCl concentration increased.

Under mild salinity stress, aboveground dry weight first increased and then decreased with increasing drought intensity. Under moderate salinity stress, aboveground dry weight initially decreased to the lowest value among the treatment groups, 0.0115 g·plant^-1^, and then increased as drought intensity increased. Under severe salinity stress, aboveground dry weight showed a gradual declining trend with increasing drought intensity. Under mild and severe drought stress, aboveground dry weight first increased and then decreased with increasing NaCl concentration, whereas under moderate drought stress, aboveground dry weight first decreased and then increased with increasing NaCl concentration.

### 3.3 Combined stress alters water status, membrane stability, and antioxidant responses

#### 3.3.1 Water retention and membrane stability are disrupted by combined stress

**Figure 4:**
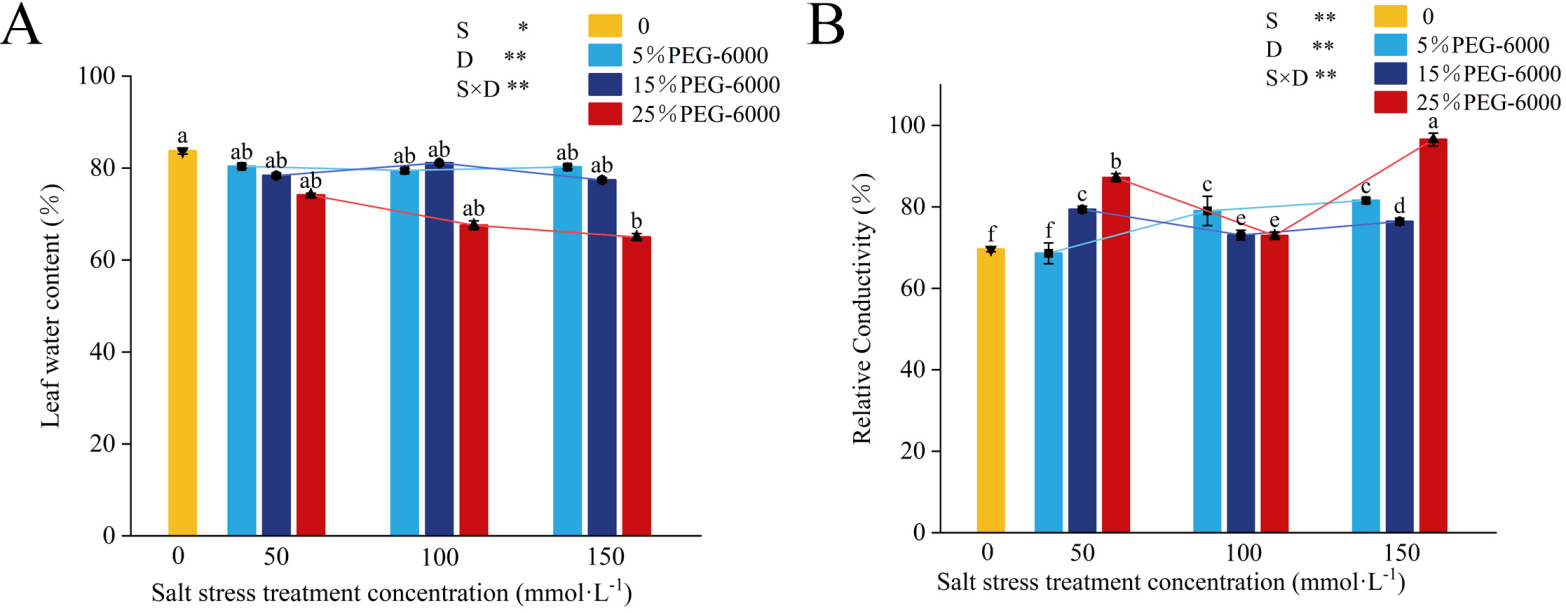
Effects of combined drought and salinity stress on the water content of alfalfa leaves and the relative electrical conductivity of seedlings. **(A)** Leaf water content, **(B)** Relative conductivity. D, drought stress; S, salinity stress; D × S, interaction between drought stress and salinity stress. Different lowercase letters above bars indicate significant differences among treatments at *P* < 0.05. *, **, and *** indicate significance at *P* < 0.05, *P* < 0.01, and *P* < 0.001, respectively; ns indicates no significant difference.

Salinity stress significantly affected leaf water content in alfalfa seedlings (*P* < 0.005), while drought stress and the drought × salinity interaction had highly significant effects on this parameter (*P* < 0.001; **Figure 4**). Under mild and severe salinity stress, leaf water content showed a slight decreasing trend with increasing drought intensity. Under moderate salinity stress, however, leaf water content first increased and then decreased as drought intensity increased. Under mild and moderate drought stress, leaf water content initially increased and then decreased with increasing NaCl concentration, whereas under severe drought stress, it decreased continuously as NaCl concentration increased.

Drought stress, salinity stress, and their interaction all had highly significant effects on relative electrical conductivity in alfalfa seedlings (*P* < 0.001; **Figure 4**). Under mild salinity stress, relative electrical conductivity increased progressively with increasing drought intensity. Under moderate salinity stress, relative electrical conductivity gradually decreased as drought intensity increased. Under severe salinity stress, relative electrical conductivity first decreased and then increased to a maximum value of 96.55% with increasing drought intensity. Under moderate and severe drought stress, relative electrical conductivity initially decreased and then increased as NaCl concentration increased. In contrast, under mild drought stress, relative electrical conductivity increased gradually with increasing NaCl concentration.

#### 3.3.2 Antioxidant enzyme activities exhibit stress-specific response patterns

**Figure 5:**
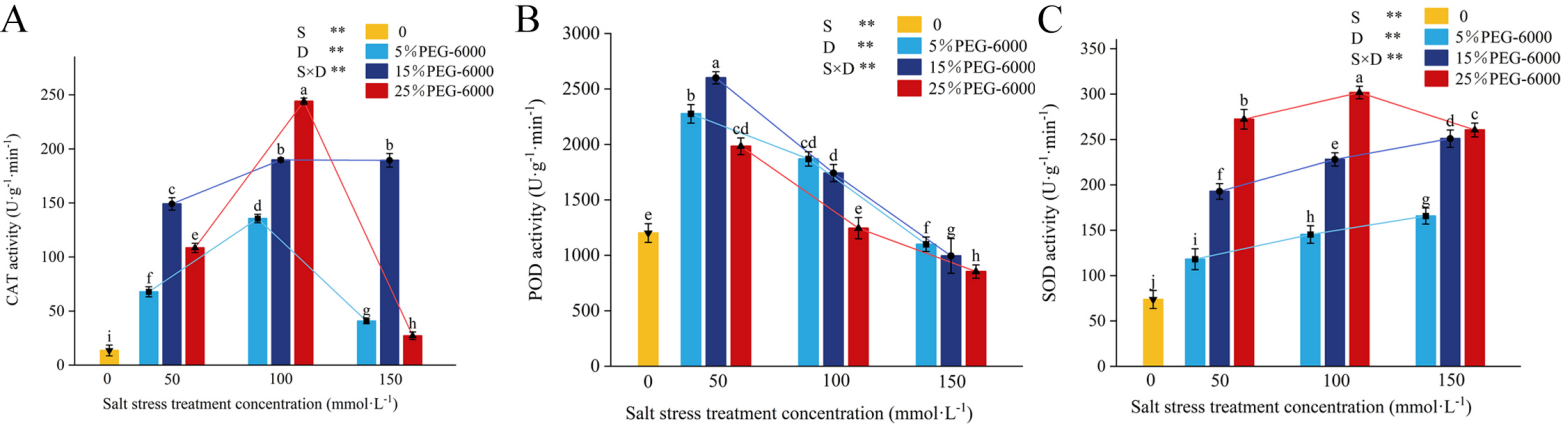
Effects of combined drought and salinity stress on the activities of catalase, peroxidase and superoxide dismutase in alfalfa. **(A)** CAT activity, **(B)** POD activity, **(C)** SOD activity. D, drought stress; S, salinity stress; D × S, interaction between drought stress and salinity stress. Different lowercase letters above bars indicate significant differences among treatments at *P* < 0.05. *, **, and *** indicate significance at *P* < 0.05, *P* < 0.01, and *P* < 0.001, respectively; ns indicates no significant difference.

Drought stress, salinity stress, and their interaction all had highly significant effects on the activities of catalase (CAT), peroxidase (POD), and superoxide dismutase (SOD) in alfalfa seedlings (*P* < 0.001; **Figure 5**). Under mild and severe salinity stress, CAT activity first increased and then decreased with increasing drought intensity. Under moderate salinity stress, however, CAT activity increased progressively as drought intensity increased. At the same drought level, CAT activity consistently showed an initial increase followed by a decrease with increasing NaCl concentration. Under mild salinity stress, POD activity first increased and then decreased with increasing drought intensity. In contrast, under moderate and severe salinity stress, POD activity declined continuously as drought intensity increased. At the same drought level, POD activity consistently decreased with increasing NaCl concentration. At the same salinity level, SOD activity increased continuously with increasing drought intensity. Under mild and moderate drought stress, SOD activity increased progressively with increasing NaCl concentration. Under severe drought stress, however, SOD activity first increased to a maximum value of 301.77 U·g^-1^·plant^-1^ and then decreased as NaCl concentration increased.

### 3.4 Trait correlations and PCA reveal coordinated responses to drought–salinity stress

**Figure 6:**
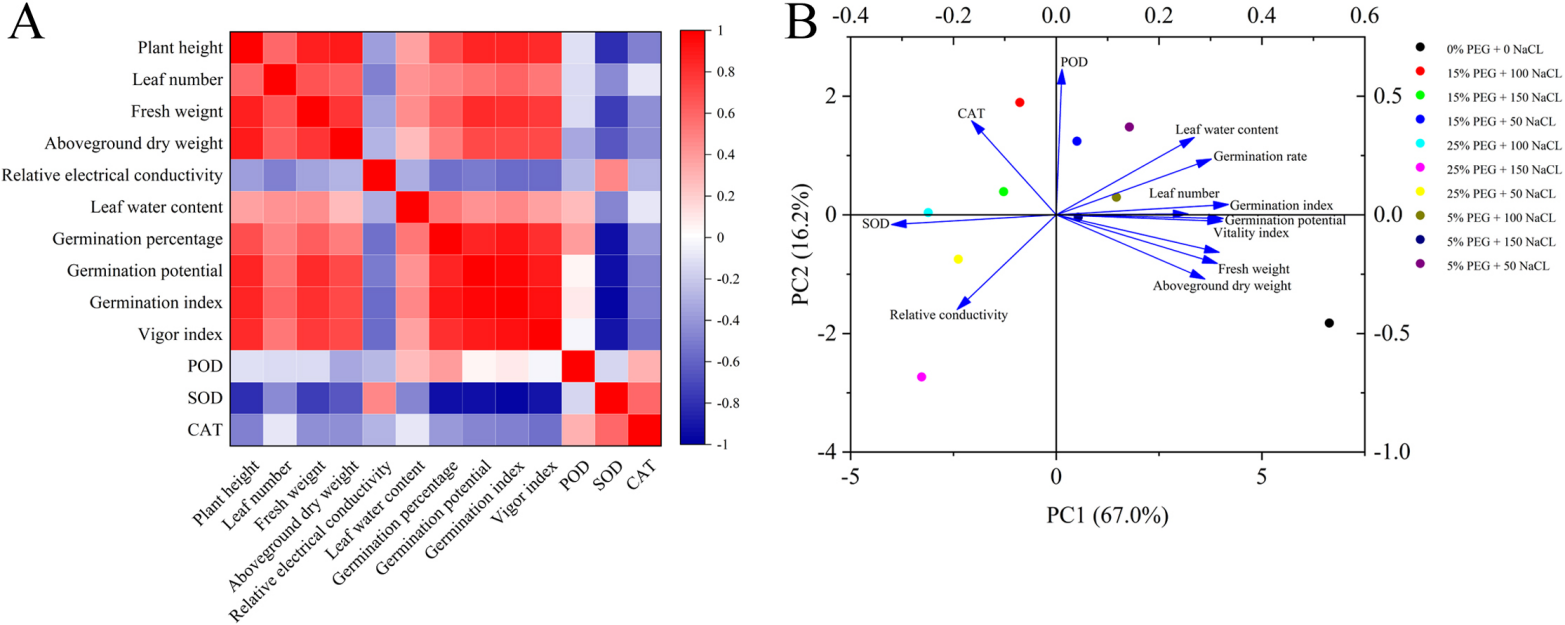
Trait correlations and principal component analysis under drought–salinity stress. **(A)** Pearson correlation heatmap among growth traits, physiological traits, enzyme activities, and germination-related traits. Red and blue colors indicate positive and negative correlations, respectively, with color intensity representing the strength of correlation. **(B)** PCA biplot showing the distribution of different PEG and NaCl treatment combinations based on all measured traits. Arrows indicate trait loadings, and points represent treatment samples. PC1 and PC2 explained 67.0% and 16.2% of the total variation, respectively, together accounting for 83.2% of the overall variation. Growth and germination-related traits were mainly associated with the positive direction of PC1, whereas relative electrical conductivity and SOD were associated with the negative direction of PC1. POD and CAT contributed mainly to variation along PC2.

Correlation and PCA analyses showed coordinated responses of alfalfa traits to combined drought and salinity stress (**Figure 6**). Germination-related traits were strongly and positively correlated with each other, and growth traits showed similar positive associations. Most germination and growth traits were positively associated with leaf water content but negatively associated with relative electrical conductivity, indicating that membrane injury was closely related to reduced seed germination and seedling growth.

PCA further showed that PC1 and PC2 explained 67.0% and 16.2% of the total variation, respectively, together accounting for 83.2% of the overall variation. Germination and growth traits were mainly distributed along the positive direction of PC1, whereas relative electrical conductivity and SOD were associated with the opposite direction. These results suggest that germination capacity, seedling growth, water retention, membrane stability, and antioxidant responses jointly contributed to treatment separation under combined drought–salinity stress.

### 3.5 Membership function analysis ranks treatment performance under combined stress

To investigate the effects of drought–salinity interaction on alfalfa seed germination and seedling growth, the membership function method was used to comprehensively evaluate stress-induced injury under combined stress treatments. As shown in **Supplementary Table 3**, the average membership values differed markedly among treatments. The control treatment had the highest average membership value, 0.82, indicating the best overall performance. Among the stress treatments, 5% PEG + 100 mmol·L^-1^ NaCl showed the highest average membership value, 0.53. As PEG and NaCl concentrations increased, the average membership value generally decreased, with the lowest value observed under 25% PEG + 150 mmol·L^-1^ NaCl, at only 0.11. According to the average membership values, the comprehensive performance of the treatments was ranked in descending order as follows: 0% PEG + 0 mmol·L^-1^ NaCl > 5% PEG + 100 mmol·L^-1^ NaCl > 5% PEG + 50 mmol·L^-1^ NaCl > 15% PEG + 50 mmol·L^-1^ NaCl > 15% PEG + 100 mmol·L^-1^ NaCl > 5% PEG + 150 mmol·L^-1^ NaCl > 15% PEG + 150 mmol·L^-1^ NaCl > 25% PEG + 100 mmol·L^-1^ NaCl > 25% PEG + 50 mmol·L^-1^ NaCl > 25% PEG + 150 mmol·L^-1^ NaCl.

**Figure 7:**
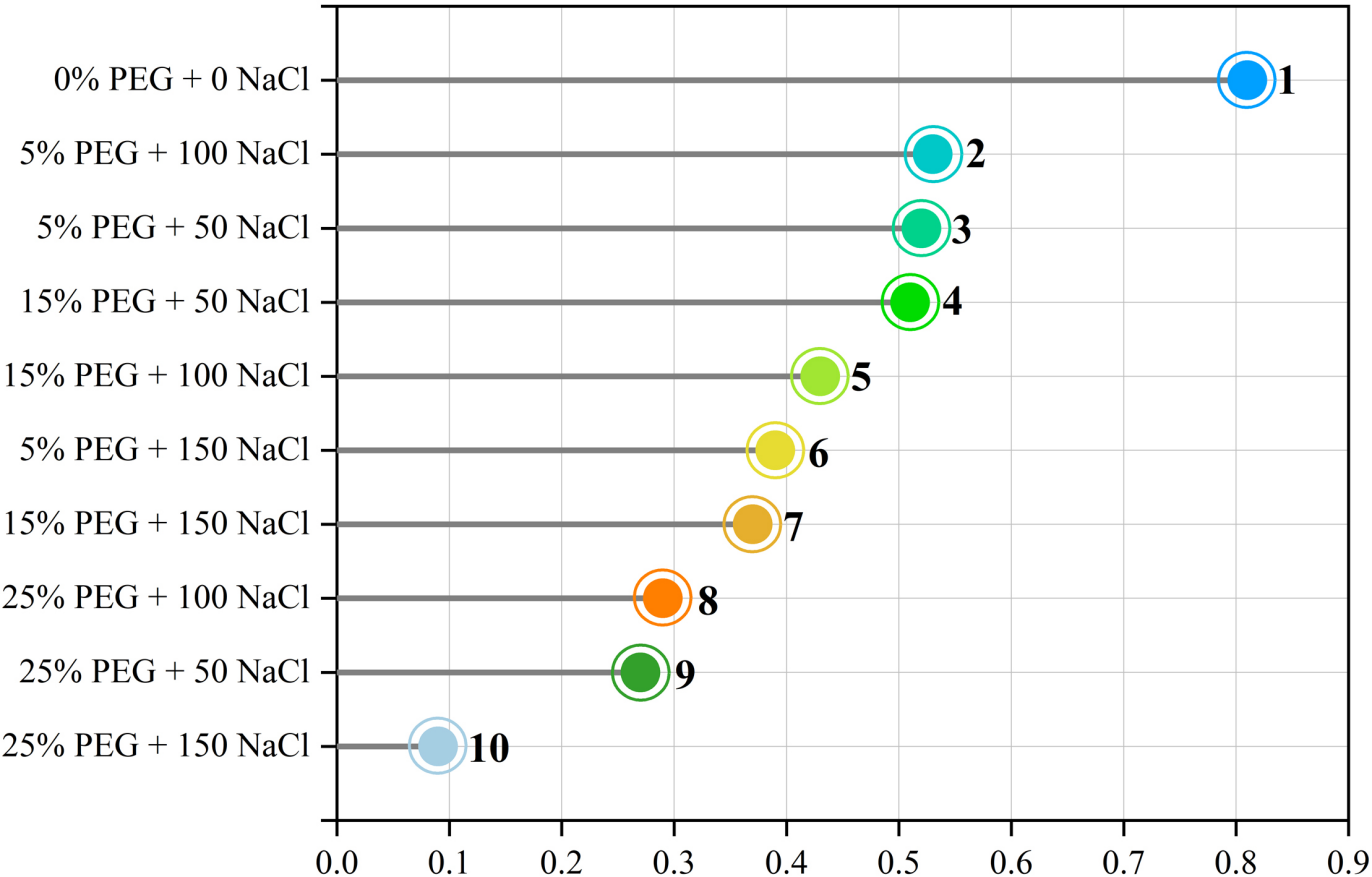
Ranking of average membership values of alfalfa under different combined drought and salinity stress treatments. Points represent the average membership value (*μ*) of each PEG–NaCl treatment, and horizontal lines indicate the corresponding ranking position. Treatments are arranged in descending order according to their average membership values. Numbers beside the points indicate the comprehensive ranking of each treatment. Higher average membership values indicate better overall performance and lower stress-induced injury, whereas lower values indicate greater injury under combined drought and salinity stress. PEG concentrations are expressed as % w/v, and NaCl concentrations are expressed as mmol·L^-1^.

The comprehensive evaluation based on the membership function method further showed that PEG and NaCl concentrations were positively associated with the degree of stress-induced injury. The average membership values of the 25% PEG treatments, ranging from 0.11 to 0.30, were lower than those of most other treatments. High-concentration combined treatments, defined as ≥15% PEG combined with ≥100 mmol·L^-1^ NaCl, caused stronger stress-induced injury (*μ* < 0.40). Although the control treatment had the highest average membership value, low-intensity combined stress treatments, especially 5% PEG combined with 50 or 100 mmol·L^-1^ NaCl, showed relatively better overall performance among the imposed stress treatments under controlled conditions (**Figure 7**). To avoid overinterpreting antioxidant enzyme activities as uniformly positive performance traits, a sensitivity analysis was conducted by excluding SOD, CAT, and POD from the membership function evaluation. The sensitivity analysis showed a generally consistent stress-injury pattern (**Supplementary Table 4**), with the control treatment ranking highest and the 25% PEG + 150 mmol·L^-1^ NaCl treatment ranking lowest. However, the ranking of the best stress treatment changed slightly, with 5% PEG + 50 mmol·L^-1^ NaCl and 5% PEG + 100 mmol·L^-1^ NaCl remaining the two highest-ranked stress treatments. These results indicate that the overall conclusion of better performance under low-intensity combined stress was robust, whereas the exact ranking of the best stress treatment should be interpreted cautiously.

### 3.6 Exploratory structural equation modeling suggests potential pathways linking stress factors to germination and aboveground dry weight

**Figure 8:**
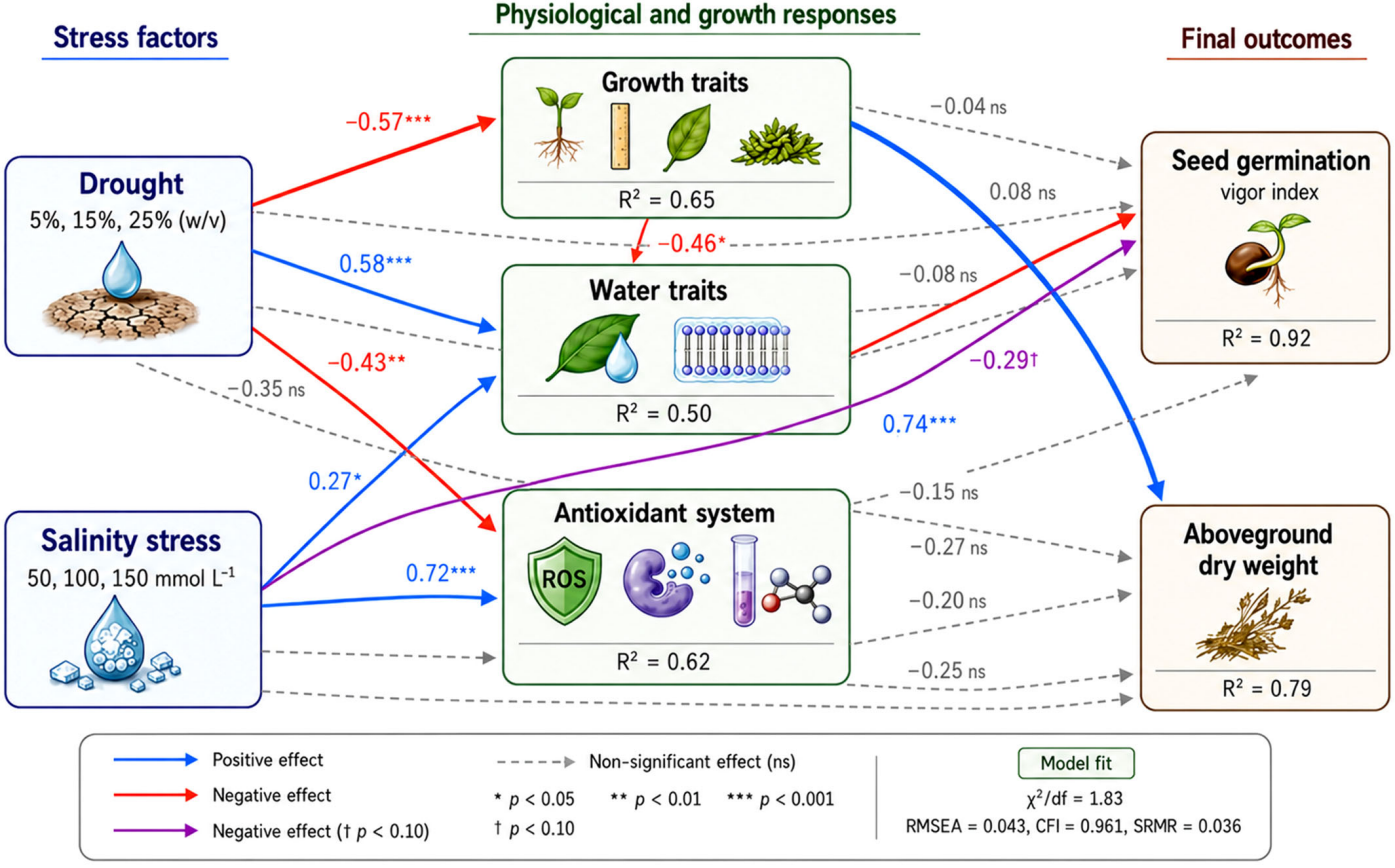
Structural equation model showing potential associations among drought stress, salinity stress, seed germination, and aboveground dry weight in alfalfa. Drought stress and salinity stress were included as exogenous variables, while growth traits, water traits, and the antioxidant system were included as intermediate variables. Seed germination and aboveground dry weight were used as final response variables. Seed germination was represented by vigor index selected based on the coefficient of variation. Blue arrows indicate positive paths, orange arrows indicate negative paths, purple arrows indicate marginally significant paths, and gray dashed arrows indicate non-significant paths. Values beside arrows are standardized path coefficients. R² values indicate the proportion of variance explained for endogenous variables. Significance levels are denoted as † *P* < 0.10, *P < 0.05, **P < 0.01, and ***P < 0.001. Model fit: χ²/df = 1.83; RMSEA = 0.043; CFI = 0.961; SRMR = 0.036.

A structural equation model was constructed to explore potential associations linking drought and salinity stress with seed germination and aboveground dry weight in alfalfa (**Figure 8**). In the model, drought stress and salinity stress were included as exogenous variables; growth traits, water traits, and the antioxidant system were included as intermediate variables; and seed germination and aboveground dry weight were included as final response variables. Seed germination was represented by the vigor index, which was selected based on the coefficient of variation. The model showed an acceptable fit, with χ²/df = 1.83, RMSEA = 0.043, CFI = 0.961, and SRMR = 0.036. Drought stress was negatively associated with growth traits and was also related to changes in water traits and the antioxidant system, whereas salinity stress was mainly associated with water traits and the antioxidant system and showed a marginal negative association with seed germination. Among the intermediate variables, growth traits showed the strongest positive association with aboveground dry weight, suggesting that seedling growth was closely linked to biomass accumulation under combined drought–salinity stress. The model explained 92% of the variation in seed germination and 79% of the variation in aboveground dry weight. Because SEM was used as an exploratory analysis, these pathways should be interpreted as potential statistical associations rather than direct causal evidence.

## 4 Discussion

With the intensification of global climate change and the continued expansion of soil salinization, drought and salinity stress have become major environmental constraints limiting the stable establishment and sustainable production of alfalfa on arid, semi-arid, and saline marginal lands. Previous studies have extensively investigated the individual effects of drought or salinity stress on seed germination, seedling growth, and physiological metabolism in alfalfa. However, under field conditions, drought stress and salt accumulation often occur simultaneously, and their interactive effects on early alfalfa establishment and the underlying physiological regulatory mechanisms remain insufficiently understood. Therefore, this study systematically evaluated the responses of alfalfa to combined drought and salinity stress in terms of seed germination, seedling growth, leaf water status, membrane stability, and antioxidant enzyme activities, aiming to provide a theoretical basis for alfalfa establishment and stress-resilient cultivation in arid and saline regions.

### 4.1 Effects of combined drought and salinity stress on seed germination

Seed germination is the first and most critical stage in the growth of alfalfa, and its sensitivity to environmental stress largely determines subsequent seedling emergence and stand establishment (Bewley, 1997; Finch-Savage and Leubner-Metzger, 2006). The results of this study showed that as PEG and NaCl concentrations increased, germination percentage, germination potential, germination index, and vigor index generally decreased, which agrees with previous studies showing that osmotic stress and salinity delay or inhibit alfalfa germination and early seedling establishment (Wang et al., 2009; Soltani et al., 2012; Castroluna et al., 2014). Seed germination performed best under the control treatment, whereas germination percentage, germination potential, germination index, and vigor index reached or approached their lowest values under the 25% PEG + 150 mmol·L^-1^ NaCl treatment. This result indicates that high-intensity combined drought–salinity stress strongly constrained the early germination process of alfalfa. In this study, 25% PEG likely created a low-water-potential environment that restricted seed imbibition, while 150 mmol·L^-1^ NaCl further imposed osmotic stress and Na^+^/Cl^-^ toxicity. Therefore, the strong inhibition of germination under this treatment may be attributed to the combined effects of PEG-induced water limitation and NaCl-induced osmotic and ionic stress. This result is generally consistent with previous studies showing that drought or salinity stress inhibits seed germination in crops and forage species. Baha (2022) reported that high concentrations of PEG and salinity stress significantly reduce seed germination and early seedling growth traits in alfalfa, and that salinity stress inhibits germination through both osmotic and ionic effects. The reductions in germination percentage, germination potential, germination index, and vigor index can be explained by several interconnected mechanisms. First, both PEG and NaCl reduce the water potential of the germination medium, thereby restricting seed imbibition and delaying or inhibiting radicle protrusion (Zhu, 2001; Verslues et al., 2006). PEG mainly simulates osmotic stress by limiting water uptake, whereas NaCl reduces external water potential and additionally imposes ionic stress during germination (Arif et al., 2020). Second, the accumulation of Na^+^ and Cl^-^ interferes with the uptake and distribution of essential ions such as K^+^ and Ca^2+^, disrupts cellular ion homeostasis, and consequently affects reserve mobilization, enzyme activation, and embryonic metabolic activity (Arif et al., 2020; Chandran et al., 2024). Third, severe osmotic and ionic stress can induce excessive reactive oxygen species accumulation, leading to membrane lipid peroxidation, membrane structural damage, and impairment of protein and enzyme activities, which ultimately reduces the germination index and vigor index (Kawaguchi et al., 2023). In addition, stress-induced changes in phytohormone regulation, especially the balance between abscisic acid and gibberellins, may further delay germination by maintaining dormancy-related responses and restricting radicle protrusion. Therefore, the decline in germination-related traits under high-intensity PEG–NaCl combinations was closely associated with reduced imbibition, osmotic stress, Na^+^/Cl^-^ toxicity, disrupted metabolic activation, oxidative stress, membrane damage, and possible hormonal regulation.

### 4.2 Effects of combined drought and salinity stress on seedling growth and biomass accumulation

Seedling growth traits, including plant height, leaf number, fresh weight, and aboveground dry weight, are key indicators of early plant establishment and directly reflect the capacity of seedlings to maintain morphogenesis and biomass accumulation under stress (Farooq et al., 2009; Cornacchione and Suarez, 2017). This study shows that seedling growth is inhibited as stress intensity increases. Leaf number reaches its lowest value, 12 leaves, under the 25% PEG + 150 mmol·L^-1^ NaCl treatment. Under mild drought stress, plant height first increases and then decreases with increasing NaCl concentration, with relatively high values occurring under some low-concentration combined stress treatments. Fresh weight under mild drought stress first increases to 0.13 g·plant^-1^ and then decreases as NaCl concentration increases. Aboveground dry weight reaches the lowest value among the treatment groups, 0.0115 g·plant^-1^, under the 15% PEG + 100 mmol·L^-1^ NaCl treatment. These results are consistent with previous studies reporting growth inhibition in alfalfa and other leguminous plants under drought or salinity stress. Previous studies show that both drought and salinity markedly suppress plant growth, with cell growth, photosynthesis, water relations, and assimilate partitioning among the earliest processes affected by drought stress and salinity stress (Chaves et al., 2009; Skirycz and Inzé, 2010). The underlying mechanisms involve three main aspects. First, drought stress reduces cellular turgor pressure and directly restricts cell division, cell elongation, and leaf expansion, thereby decreasing plant height and leaf number (Yang et al., 2021). Second, salt ion accumulation causes Na^+^/K^+^ imbalance and affects protein synthesis, enzyme activity, and photosynthesis through osmotic stress and ionic toxicity, ultimately reducing fresh weight and dry matter accumulation (Munns and Tester, 2008; Zhou H. et al., 2024). Third, combined drought and salinity stress simultaneously disrupts plant water balance, ion homeostasis, and redox balance, while increasing the metabolic costs required for osmotic adjustment, ion compartmentalization, and antioxidant defense. As a result, more assimilates are diverted to stress protection rather than growth and development (Angon et al., 2022). Thus, high PEG–NaCl combinations exerted a strong combined inhibitory effect on alfalfa seedling growth.

### 4.3 Effects of combined drought and salinity stress on leaf water status and membrane stability

Leaf water content and relative electrical conductivity are important indicators of plant water status and cell membrane stability under osmotic and ionic stress (Bartels and Sunkar, 2005; Zhang and Shi, 2018). The results of this study showed that under mild and moderate drought stress, leaf water content first increased and then decreased with increasing NaCl concentration, whereas under severe drought stress, it decreased continuously. Under severe salinity stress, relative electrical conductivity first decreased and then increased to a maximum value of 96.55% with increasing drought intensity. These results are consistent with previous findings that drought and salinity stress aggravate plant injury by reducing tissue water content and increasing membrane permeability. Previous studies have shown that drought stress decreases leaf water relations, membrane stability, and photosynthetic activity, accompanied by increased reactive oxygen species accumulation, lipid peroxidation, and membrane injury (Farooq et al., 2009); salinity stress disrupts cellular homeostasis through the combined effects of osmotic stress, ion toxicity, and oxidative stress (Abid et al., 2018; Kylyshbayeva et al., 2024). The decline in leaf water content and the increase in relative electrical conductivity may be explained by three mechanisms. First, PEG treatment can induce a low-water-potential environment in the culture system, thereby imposing osmotic stress and restricting root water uptake, which results in insufficient water supply to leaves (Fan and Blake, 1997; Kylyshbayeva et al., 2024). Second, NaCl-induced salinity aggravates ion toxicity and osmotic stress, disturbs ion homeostasis, and damages membrane structures, thereby increasing electrolyte leakage (Isayenkov and Maathuis, 2019; Hao et al., 2021). Third, prolonged stress induces reactive oxygen species accumulation and promotes membrane lipid peroxidation, leading to reduced membrane integrity and, ultimately, increased relative electrical conductivity (Kesawat et al., 2023).

### 4.4 Effects of combined drought and salinity stress on antioxidant enzyme activities

Antioxidant enzymes, including superoxide dismutase (SOD), catalase (CAT), and peroxidase (POD), are key components of the plant defense system and play essential roles in scavenging reactive oxygen species and maintaining redox homeostasis under abiotic stress (Mahajan and Tuteja, 2005; Guo et al., 2022). The results show that under the same salinity level, SOD activity generally increases as drought intensity increases. Under severe drought stress, SOD activity first increases to a peak value and then decreases with increasing NaCl concentration. CAT activity first increases and then decreases with increasing drought intensity under mild and severe salinity stress, whereas it increases progressively under moderate salinity stress. POD activity exhibits a response pattern distinct from those of SOD and CAT: under mild salinity stress, POD activity first increases and then decreases with increasing drought intensity, whereas under moderate and severe salinity stress, it decreases continuously as drought intensity increases. Previous studies show that abiotic stress often induces excessive reactive oxygen species (ROS) accumulation, while antioxidant enzymes such as SOD, CAT, and POD constitute an important defense system for ROS detoxification, redox homeostasis maintenance, and stress adaptation in plants (Sharma et al., 2019; Kesawat et al., 2023). The differential changes in antioxidant enzyme activities can be explained by several mechanisms. First, mild to moderate stress induces moderate ROS signaling, thereby activating the expression and activity of defense enzymes such as SOD, CAT, and POD and enhancing stress adaptation (Mittler, 2017). Second, high-intensity combined stress leads to excessive ROS accumulation, resulting in oxidative damage, protein oxidation, enzyme structural impairment, or disruption of substrate supply, which may reduce the activity of some enzymes (Sewelam et al., 2016; Sachdev et al., 2021). Third, different antioxidant enzymes occupy distinct functional positions in the ROS-scavenging pathway, and therefore their responses are not necessarily synchronized. SOD mainly catalyzes the conversion of superoxide radicals into H_2_O_2_, whereas CAT and POD further participate in H_2_O_2_ detoxification (Sharma et al., 2019).

### 4.5 Integrated evaluation of key regulatory traits in alfalfa under combined drought and salinity stress

Correlation analysis, PCA, membership function analysis, and structural equation modeling jointly revealed the integrated response pattern of alfalfa under combined drought and salinity stress (Li et al., 2021; Wang et al., 2023). Germination and growth traits were positively associated with each other, whereas relative electrical conductivity was negatively correlated with most germination and growth traits, indicating that membrane injury was closely linked to reduced early establishment. PCA further supported this pattern, with PC1 and PC2 explaining 67.0% and 16.2% of the total variation, respectively. These results suggest that germination capacity, seedling growth, water status, membrane stability, and antioxidant responses were the major trait dimensions separating alfalfa responses to different drought–salinity combinations.

The membership function analysis showed that the control treatment had the highest average membership value, while low-intensity combined stress treatments, especially 5% PEG combined with 50 or 100 mmol·L^-1^ NaCl, showed relatively better performance among the imposed stress treatments under controlled conditions. Exploratory structural equation modeling further suggested potential statistical associations among stress factors, intermediate trait groups, seed germination, and aboveground dry weight. Drought stress was negatively associated with growth traits and was related to changes in water-related and antioxidant responses, whereas salinity stress was mainly associated with water traits and the antioxidant system and showed a marginal negative association with seed germination. Growth traits showed the strongest association with aboveground dry weight. Together, these findings suggest that early alfalfa establishment under combined drought and salinity stress may be associated with coordinated changes in germination capacity, seedling growth, membrane stability, water status, and antioxidant responses.

The relatively better performance under low-intensity combined stress may be related to limited adaptive responses, such as osmotic adjustment, antioxidant regulation, and maintenance of membrane stability, whereas severe combined stress may exceed the regulatory capacity of seedlings by simultaneously inducing water limitation, ion toxicity, and oxidative damage (Yang et al., 2021; Angon et al., 2022; Cao et al., 2023). Therefore, high-intensity drought–salinity combinations should be avoided during germination and early seedling growth. However, because the PEG–NaCl system under controlled conditions may not fully represent field environments, future studies integrating field validation, multiple alfalfa cultivars, and physiological–molecular analyses are needed to further clarify the tolerance mechanisms and practical applicability of these findings. In addition, the osmotic potentials of PEG-6000 and PEG–NaCl mixtures were estimated rather than directly measured, and substrate electrical conductivity and PEG concentration in the pot medium were not continuously monitored during the 20-day stress period. Thus, the actual osmotic and ionic conditions experienced by seedlings, particularly in the pot-substrate system, may have differed from the nominal treatment concentrations. Furthermore, key physiological and biochemical indicators, including Na^+^, K^+^, Cl^-^, osmolytes, ROS, MDA, proline, and hormone concentrations, were not directly measured. Therefore, the roles of ion toxicity, osmotic adjustment, oxidative damage, and ABA/GA-mediated hormonal regulation require further validation in future studies.

## 5 Conclusions

This study demonstrated that drought stress, salinity stress, and their interaction significantly affected seed germination, early seedling growth, and physiological responses of alfalfa under controlled conditions. Increasing PEG and NaCl concentrations generally suppressed germination performance, reduced seedling growth and biomass accumulation, decreased leaf water retention, and impaired membrane stability, with the strongest inhibition observed under the 25% PEG + 150 mmol·L^-1^ NaCl treatment. Multivariate analyses and membership function evaluation indicated that alfalfa responses to combined drought and salinity stress were closely associated with germination capacity, seedling growth, water status, membrane stability, and antioxidant responses. Among the imposed stress treatments under controlled conditions, low-intensity combined stress treatments, especially 5% PEG combined with 50 or 100 mmol·L^-1^ NaCl, showed relatively better overall performance, whereas high-intensity stress caused severe injury. Exploratory structural equation modeling suggested potential statistical associations among drought and salinity stress, growth traits, water traits, antioxidant responses, seed germination, and aboveground dry weight, with growth traits being closely linked to aboveground dry weight.

## Data Availability

The raw data supporting the conclusions of this article will be made available by the authors, without undue reservation.
