# Supplementary material for: Drought–salinity interaction suppresses seed germination and seedling growth in alfalfa by altering water status, membrane stability, and antioxidant responses
Source: Front Plant Sci. 2026 Jul 28;17:1887737. doi: 10.3389/fpls.2026.1887737 (PMC13460708; doi:10.3389/fpls.2026.1887737)
Supplement: Supplementary file 1 [file Table1.docx]

Supplementary Material

# 1 Supplementary Tables

## 1.1 Supplementary Table S1. Estimated osmotic potentials of PEG-6000, NaCl, and PEG–NaCl mixed treatment solutions at 25°C.

| PEG-6000 concentration | Estimated ΨPEG (MPa) | NaCl concentration | Estimated ΨNaCl (MPa) | Estimated combined ΨPEG+NaCl (MPa) |
| --- | --- | --- | --- | --- |
| 0% | 0.000 | 0 mmol·L^-1^ | 0.000 | 0.000 |
| 5% | -0.050 | 50 mmol·L^-1^ | -0.248 | -0.298 |
| 5% | -0.050 | 100 mmol·L^-1^ | -0.496 | -0.546 |
| 5% | -0.050 | 150 mmol·L^-1^ | -0.744 | -0.794 |
| 15% | -0.295 | 50 mmol·L^-1^ | -0.248 | -0.543 |
| 15% | -0.295 | 100 mmol·L^-1^ | -0.496 | -0.791 |
| 15% | -0.295 | 150 mmol·L^-1^ | -0.744 | -1.039 |
| 25% | -0.735 | 50 mmol·L^-1^ | -0.248 | -0.983 |
| 25% | -0.735 | 100 mmol·L^-1^ | -0.496 | -1.230 |
| 25% | -0.735 | 150 mmol·L^-1^ | -0.744 | -1.478 |

Note: ΨPEG was estimated using the empirical equation of Michel and Kaufmann (1973): ΨPEG = [−(1.18 × 10^⁻²^)C − (1.18 × 10^⁻⁴^)C² + (2.67 × 10^⁻⁴^)CT + (8.39 × 10^⁻⁷^)C^²^T] × 0.1, where C is PEG-6000 concentration expressed as g·kg^⁻¹^ water and T is temperature in °C. ΨNaCl was estimated using the van’t Hoff equation: ΨNaCl = −iCRT, where i = 2 for NaCl, C is molar concentration, R = 0.008314 L·MPa·mol^⁻¹^·K^⁻¹^, and T = 298.15 K. The combined osmotic potential was approximated as ΨPEG + ΨNaCl. These values are estimated reference values rather than directly measured osmotic potentials.

## 1.2 Supplementary Table S2. Coefficient of variation ranking of seed germination indices.

|  | Mean | SD | CV (%) | Order |
| --- | --- | --- | --- | --- |
| Germination Percentage | 0.50 | 0.35 | 69.65 | 4 |
| Germination Potential | 0.27 | 0.28 | 104.40 | 2 |
| Germination Index | 35.62 | 31.81 | 89.29 | 3 |
| Vigor Index | 270.14 | 322.72 | 119.46 | 1 |

# Note: SD, standard deviation; CV, coefficient of variation. CV was calculated using unrounded original data.

## 1.3 Supplementary Table S3. Comprehensive evaluation of alfalfa performance under drought–salinity interaction stress.

| Treatment | Germination potential | Germination percentage | Germination index | Vigor index | Leaf number | | Plant height | Aboveground dry weight | | Fresh weight | Leaf water content | | Relative electrical conductivity | | SOD | CAT | | POD | | Average membership value | | | Order | |
| --- | --- | --- | --- | --- | --- | --- | --- | --- | --- | --- | --- | --- | --- | --- | --- | --- | --- | --- | --- | --- | --- | --- | --- | --- |
| 0% PEG + 0 NaCl | 1.00 | 1.00 | 1.00 | 1.00 | | 1.00 | 1.00 | 1.00 | 1.00 | | | 1.00 | | 0.97 | 0.24 | | 0.00 | | 0.46 | | 0.82 | 1 | |  |
| 5% PEG + 50 NaCl | 0.88 | 0.56 | 0.61 | 0.68 | | 0.34 | 0.35 | 0.15 | 0.14 | | | 0.67 | | 1.00 | 0.39 | | 0.12 | | 0.88 | | 0.52 | 3 | |  |
| 5% PEG + 100 NaCl | 0.74 | 0.68 | 0.54 | 0.31 | | 0.38 | 0.47 | 0.43 | 0.35 | | | 0.62 | | 0.63 | 0.48 | | 0.50 | | 0.72 | | 0.53 | 2 | |  |
| 5% PEG + 150 NaCl | 0.77 | 0.29 | 0.38 | 0.26 | | 0.43 | 0.26 | 0.30 | 0.19 | | | 0.66 | | 0.54 | 0.54 | | 0.00 | | 0.42 | | 0.39 | 6 | |  |
| 15% PEG + 50 NaCl | 0.74 | 0.28 | 0.39 | 0.30 | | 0.53 | 0.40 | 0.35 | 0.23 | | | 0.55 | | 0.62 | 0.63 | | 0.56 | | 1.00 | | 0.51 | 4 | |  |
| 15% PEG + 100 NaCl | 0.58 | 0.20 | 0.30 | 0.10 | | 0.63 | 0.00 | 0.00 | 0.00 | | | 0.73 | | 0.80 | 0.75 | | 0.77 | | 0.67 | | 0.43 | 5 | |  |
| 15% PEG + 150 NaCl | 0.26 | 0.00 | 0.12 | 0.05 | | 0.56 | 0.24 | 0.26 | 0.14 | | | 0.50 | | 0.72 | 0.82 | | 0.76 | | 0.38 | | 0.37 | 7 | |  |
| 25% PEG + 50 NaCl | 0.16 | 0 | 0.06 | 0.02 | | 0.34 | 0.18 | 0.10 | 0.00 | | | 0.36 | | 0.34 | 0.89 | | 0.36 | | 0.76 | | 0.28 | 9 | |  |
| 25% PEG + 100 NaCl | 0.01 | 0 | 0.00 | 0.00 | | 0.38 | 0.02 | 0.15 | 0 | | | 0.00 | | 0.81 | 1.00 | | 1.00 | | 0.48 | | 0.30 | 8 | |  |
| 25% PEG + 150 NaCl | 0.00 | 0 | 0 | 0.00 | | 0.00 | 0.06 | 0.15 | 0.01 | | | 0 | | 0.00 | 0.86 | | 0 | | 0.33 | | 0.11 | 10 | |  |

Note: PEG concentrations are expressed as % (w/v), and NaCl concentrations are expressed as mmol·L^⁻¹^.

## 1.4 Supplementary Table S4. Sensitivity analysis of membership function ranking after excluding antioxidant enzyme activities.

| Treatment | Average membership value after excluding SOD, CAT, and POD | Order |
| --- | --- | --- |
| 0% PEG + 0 NaCl | 0.997 | 1 |
| 5% PEG + 50 NaCl | 0.538 | 2 |
| 5% PEG + 100 NaCl | 0.515 | 3 |
| 15% PEG + 50 NaCl | 0.439 | 4 |
| 5% PEG + 150 NaCl | 0.408 | 5 |
| 15% PEG + 100 NaCl | 0.334 | 6 |
| 15% PEG + 150 NaCl | 0.285 | 7 |
| 25% PEG + 50 NaCl | 0.156 | 8 |
| 25% PEG + 100 NaCl | 0.137 | 9 |
| 25% PEG + 150 NaCl | 0.022 | 10 |
